# Monitoring the response of volcanic CO_2_ emissions to changes in the Los Humeros hydrothermal system

**DOI:** 10.1038/s41598-021-97023-x

**Published:** 2021-09-09

**Authors:** Anna Jentsch, Walter Duesing, Egbert Jolie, Martin Zimmer

**Affiliations:** 1 Helmholtz Centre Potsdam German Research Centre for Geosciences GFZ, Telegrafenberg, 14473 Potsdam, Germany; 2 Institute of Geosciences, University of Potsdam, Karl-Liebknecht-Str. 24-25, 14476 Potsdam, Germany

**Keywords:** Geochemistry, Geology, Geophysics, Volcanology, Energy and society

## Abstract

Carbon dioxide is the most abundant, non-condensable gas in volcanic systems, released into the atmosphere through either diffuse or advective fluid flow. The emission of substantial amounts of CO_2_ at Earth’s surface is not only controlled by volcanic plumes during periods of eruptive activity or fumaroles, but also by soil degassing along permeable structures in the subsurface. Monitoring of these processes is of utmost importance for volcanic hazard analyses, and is also relevant for managing geothermal resources. Fluid-bearing faults are key elements of economic value for geothermal power generation. Here, we describe for the first time how sensitively and quickly natural gas emissions react to changes within a deep hydrothermal system due to geothermal fluid reinjection. For this purpose, we deployed an automated, multi-chamber CO_2_ flux monitoring system within the damage zone of a deep-rooted major normal fault in the Los Humeros Volcanic Complex (LHVC) in Mexico and recorded data over a period of five months. After removing the atmospheric effects on variations in CO_2_ flux, we calculated correlation coefficients between residual CO_2_ emissions and reinjection rates, identifying an inverse correlation of ρ = − 0.51 to − 0.66. Our results indicate that gas emissions respond to changes in reinjection rates within 24 h, proving an active hydraulic communication between the hydrothermal system and Earth’s surface. This finding is a promising indication not only for geothermal reservoir monitoring but also for advanced long-term volcanic risk analysis. Response times allow for estimation of fluid migration velocities, which is a key constraint for conceptual and numerical modelling of fluid flow in fracture-dominated systems.

## Introduction

Worldwide, a large number of caldera-hosted geothermal systems are located along volcanic arcs, such as the Los Humeros Volcanic caldera (LHVC) in the Trans-Mexican Volcanic Belt (Mexico) or Onikobe in the Honshu Arc (Japan). Such geothermal systems contain a vast potential of geothermal energy^1,2^. Calderas are very complex, large-scale^3^ geological structures that provide elevated heat flow within relatively shallow depths (< 2 km) lasting for several thousands of years. This makes them a preferred target in geothermal exploration^4–6^. Their structural evolution is of particular interest, since a comprehensive understanding of the localization of permeable fluid pathways, as well as of their structural controls, are key objectives for the successful utilization of geothermal energy^7,8^. Deep-rooted fault zones and fracture networks connecting geothermal reservoirs to Earth’s surface channel vast amounts of hydrothermal fluids^9–12^. In undisturbed conditions, migrating fluids can form stable and long-lasting geothermal surface manifestations such as fumaroles or hot springs, which provide valuable information about reservoir conditions^13,14^. However, volcano-tectonic activity or the development of geothermal resources for power generation can change this equilibrium.

A sustainable field management requires comprehensive monitoring of physical and chemical changes in geothermal reservoirs during production and reinjection of fluids for a timely reaction to pressure decline and temperature depletion, respectively^15^. Reinjection of extracted geothermal fluids (brine) into the feed, or loss zones, of a geothermal system is essential to maintain reservoir pressure and fluid recharge, control subsidence and avoid contamination of local ground water^16,17^. This requires site-specific strategies for reinjection at suitable locations, thus avoiding any interference in the production zone by thermal breakthrough, mineral precipitation or induced seismicity^18,19^.

For the first time, in this study we investigated the relationship between induced CO_2_ flux variability and changes in reinjection rates in a geothermal system. We deployed a multi-chamber CO_2_ flux monitoring system within the damage zone of a large normal fault crossing the Los Humeros geothermal field, in combination with an on-site meteorological station. After we quantified the variations in CO_2_ flux induced by atmospheric parameters, we used the time series of residual CO_2_ emissions to calculate their correlation coefficients with daily reinjection rates. The results show an inverse correlation between the two parameters within a time window of ≤ 24 h. We additionally discuss further unmonitored variables and nonlinear processes that could have a potential impact on CO_2_ variations.

Our approach combines geochemical surface data and physical subsurface data in order to develop a thorough understanding of induced fluid migration from the geothermal reservoir along specific flow paths to the Earth’s surface.

### Los Humeros geothermal system

**Figure 1:**
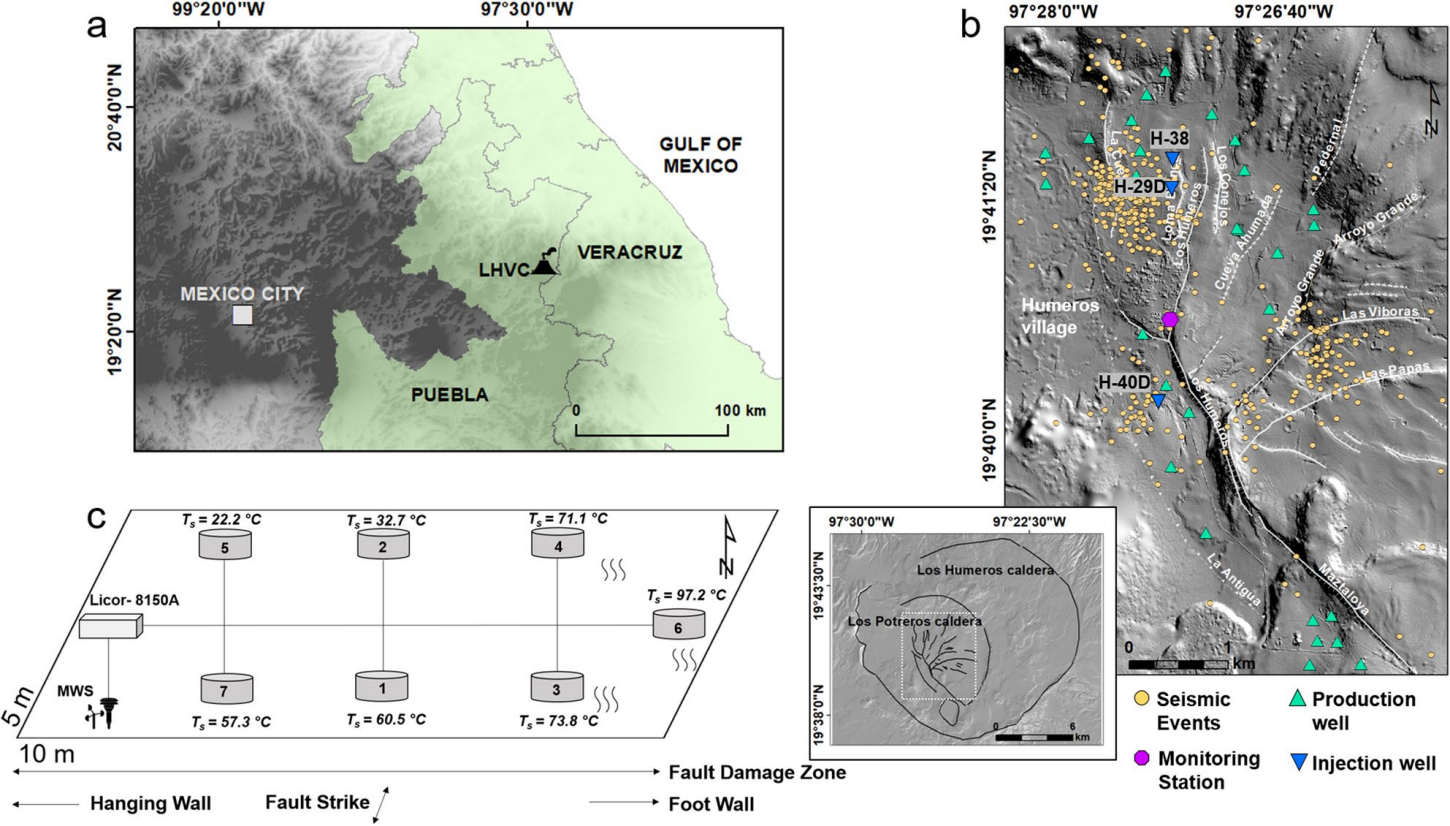
(**a**) Location of the Los Humeros Volcanic Complex (LHVC) on the border of the federal states Puebla and Veracruz on a 120 m-resolution digital elevation model (DEM), available at https://www.inegi.org.mx/app/geo2/elevacionesmex/. (**b**) Overview of the main production field of Los Humeros (Installed capacity 93.9 MWe^21^) on a shaded relief image obtained from a 1 m-resolution DEM^22^. White solid and dashed lines illustrate known and inferred faults, respectively. Orange circles represent locations of seismic events from September 2017 to September 2018^23^. The inset map, illustrated by the white dashed rectangle, shows the extent of the LHVC and Los Potreros calderas hosting the active geothermal field. The maps were generated using ArcGIS 10.4.1 software. (**c**) Setup of the CO_2_ monitoring array within the fault damage zone of the Los Humeros fault. Ts values indicate ground temperatures measured during the initial site selection survey, while black arrows show the direction and extension of fault geometry parameters.

The Los Humeros Volcanic Complex (LHVC) is the result of the largest caldera-forming eruption in the 1000 km-long Trans-Mexican Volcanic Belt (TMVB) (Fig. 1a). Ref. ^20^ determined that the LHVC contains a volume of 290 km^3^,1b).

The LPC hosts the high-temperature (~ 380 °C at > 2100 m below surface), two-phase, liquid-dominated Los Humeros geothermal reservoir, which is controlled by secondary permeability, e.g., faults and fractures^24^. The reservoir fluids are rich in CO_2_ and other non-condensable gases^25,26^. Helium isotopic ratios, determined in fluid samples from wells, are characteristic of the sub-continental mantle and suggest heat supply from an active magmatic system^27^.

Both volcanic activity and regional tectonics are the dominant forces on the structural architecture of the caldera. The volcano-tectonic interaction is responsible for the fault system’s complexity^28^. Formation permeability of the andesitic to basaltic geothermal reservoir is very low (K < 10^–16^ m^2^)^29^. Thus, fluid migration is mainly favoured by fault zones and fracture networks that cut through the overlying volcanic rocks. Macro-fracture permeability, characterized by regional faults in the geothermal system, can either enhance or impede fluid migration through the formation of fluid conduits or barriers^28^.

Two of the most distinctive faults, the Los Humeros and Maztaloya faults, merge in the central part of the geothermal reservoir. Several smaller fault strands in the northern part of the Los Humeros fault (e.g., La Cuesta, Loma Blanca, Los Conejos) build a horsetail structure forming a wide zone of substantial hydrothermal alteration^28^. Within this structure, increased CO_2_ degassing and multiple thermal anomalies with ground temperatures up to 92 °C at 50 cm depth^30,31^ are observed. The Los Humeros fault is a deep-seated, permeable fault zone facilitating the migration of geothermal fluids. It is therefore targeted by both production and reinjection wells (Fig. 1b). This makes the fault an ideal location in which to study the response of natural gas emissions at Earth’s surface to reinjection-induced changes in the geothermal reservoir.

## Data and methods

We installed an LI-COR Li-8100 automated soil CO_2_ flux monitoring system with seven accumulation chambers on an area of 50 m^2^ in combination with an on-site weather station (MWS 9-5; Fig. 1c, Fig. S1; Table S1 in the supplemental material) for continuous observation of air temperature, barometric pressure, air humidity, precipitation, and wind speed and direction. The monitoring site was chosen to be well-linked to a fault displaying thermal anomalies and increased degassing, distinctive from background CO_2_ emissions. For this reason, CO_2_ flux and ground temperatures at 50 cm depth were measured before the monitoring network was deployed.

The vegetation cover of the study site is sparse and characterized by irregular tufts of grass, small shrubs, cacti and agaves. Several pine trees border the eastern side of the monitoring area; thus, we cannot exclude the influence of root respiration^32^ on CO_2_ flux at stations 3, 4 and 6 (Fig. S1). Most of the study site lacks a surficial organic layer due to anomalous ground temperatures (T_s_ > 30 °C) that control the distribution of argillic alteration, as recognized by clay minerals such as kaolinite^33^. The subsurface consists of alternating layers of unconsolidated pumice and scoria lapilli, which have a wide range of grain sizes^34^. Each monitoring station measured soil CO_2_ flux on an hourly basis following the accumulation chamber method^11,35,36^ over a period of five months, from April to September 2018. The setup with multiple accumulation chambers was chosen due to the following advantages compared to single monitoring stations: (i) monitoring CO_2_ fluxes of different magnitudes and origins (hydrothermal/biogenic), (ii) continuous datasets for benchmarking between individual sampling sites, (iii) robust quantitative assessment of the influence of meteorological parameters on gas flux, and (iv) understanding the spatial variability of CO_2_ flux on small areas in relation to (sub)surface heterogeneities (fault zone architecture, soil type, alteration).

The geothermal reservoir has been the site of more than 60 wells in the past 40 years^37^. Infield reinjection began five years after the commercial utilization of geothermal energy started in 1990^38^. Currently, three wells with depths of 2200 m are used to reinject geothermal fluids at a total average rate of 171 t/h and 28 production wells with a production of 663 t/h (2018, unpublished data from CFE Comisión Federal de Electricidad; Fig. 1b). The amount of reinjected fluids at Los Humeros depends on the available brine, which has always been low compared to the amount of produced fluids (liquid and steam). An increase in reinjection rates usually occurs in response to an observed decline in productivity, but it is a balancing act to inject the appropriate amount of fluids without decreasing production enthalpies. In recent years, geothermal operators at Los Humeros have incorporated condensing technologies into the power units to increase the amount of fluids used for reinjection (personal communication). Daily reinjection and monthly production rates were provided by the geothermal power plant operator CFE.

From September 2017 to September 2018, seismic activity was continuously monitored using 25 broadband and 20 short-period stations across the Los Humeros geothermal field^23^. Three distinct clusters of induced/natural seismicity (*M*_LV_ ≤ 2.1) are indicated in the vicinity of production and reinjection wells (Fig. 1b) at depths between 1 and 3.5 km, corresponding to the depth of the geothermal reservoir. During CO_2_ flux monitoring, the magnitude of seismic events (124 in total) ranged from − 0.61 to 2.1 M_LV_ (unpublished data).

**Figure 2:**
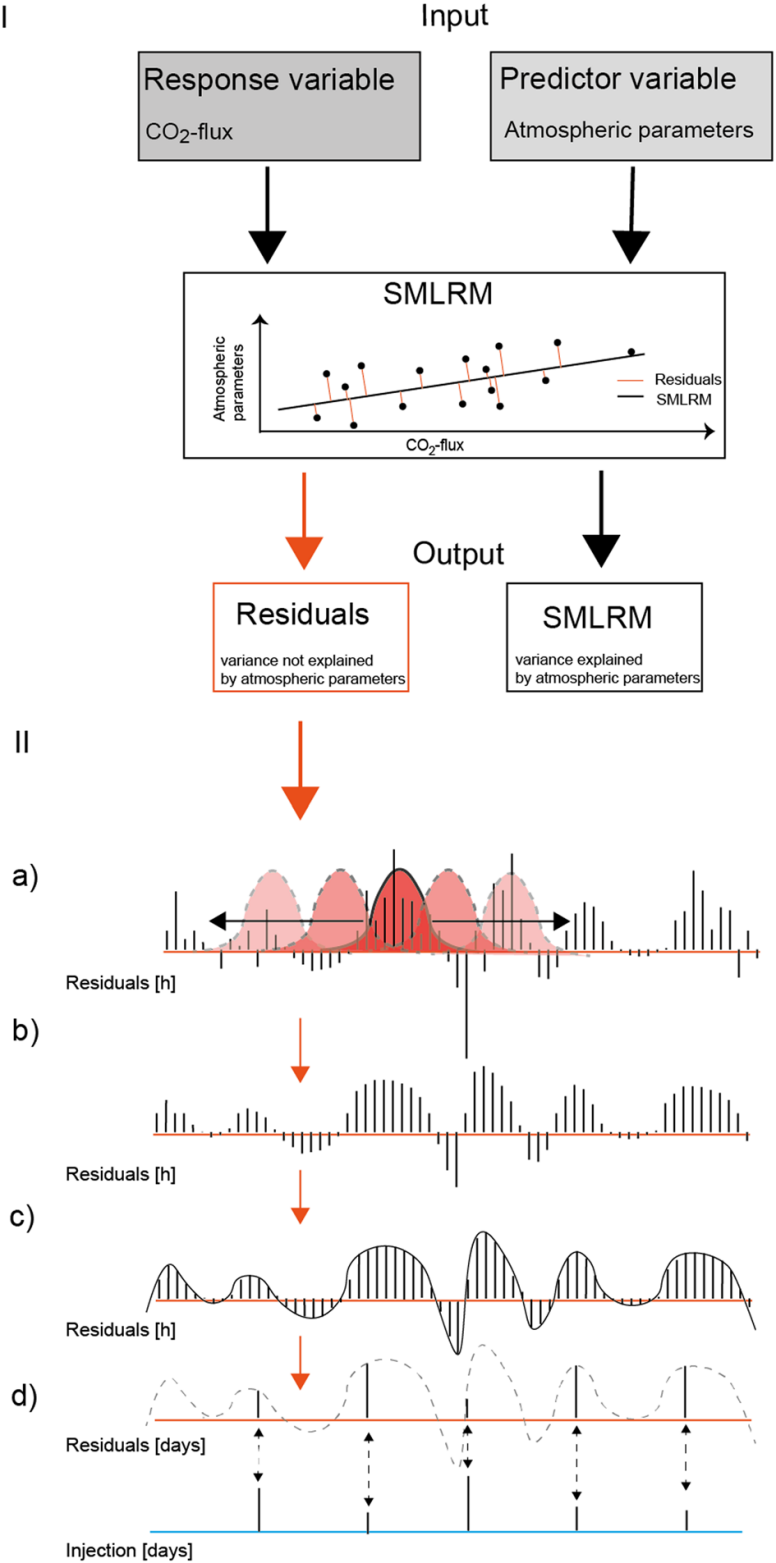
Workflow of the statistical analysis of the monitored data. Part I shows a flow chart of the stepwise multiple linear regression model (SMLRM). The SMLRM requires two input variables, the predictor variable, and the response variable. While the predictor variable usually consists of a multidimensional data set, as in our case atmospheric parameters (e.g., air temperature, wind speed), the response variable is a one-dimensional data set (measured CO_2_ flux). The output of the SMLRM is a linear regression model that represents the variability of the response variable according to the predictor variables. The remaining residuals represent the variability of the response variable which is not explained by the predictor variables. In this study we focused on the residuals. Part II visualizes the resampling of the residuals. (**a**) Describes the application of the 24-h Gaussian filter running in both, forward and reverse directions, (**b**) to smooth the hourly measured CO_2_ fluxes. (**c**) Shows the application of a shape-preserving piecewise cubic interpolation and finally, (**d**) resampling of hourly resolved residuals of the SMLRM to daily resolution. Consequently, we obtain daily resolved residuals that can be correlated with daily reinjection rates.

The collected datasets were statistically analysed using MATLAB software version R2019b. The statistical correlations among (i) the CO_2_ flux chambers and (ii) each CO_2_ flux chamber and the meteorological parameters were calculated using a spearman’s rank correlation matrix. Removing atmospheric effects on CO_2_ flux data is crucial for determining the influence of endogenous processes on CO_2_ variations^37–41^. This was conducted by computing a stepwise multiple linear regression model (SMLRM)^42^ for each CO_2_-flux time series. For the SMLRM, we excluded all data gaps, thereby reducing the multidimensional data set from 3552 to 2971 data points. The stepwise regression is a systematic method that describes the relationship between the response variable (CO_2_ flux) and the predictor variables (atmospheric parameters) by first adding and then removing one variable at a time to the model. The final model is reached when the residual sum of squares (R^2^) no longer changes. The adjusted R^2^ value explains the amount of variation computed by the linear regression model. The p-value is a criterion which defines whether variables should be removed or added to the model, with the default threshold set to 0.05. A p-value below 0.05 is usually considered to be a sufficient rejection of the null hypothesis^43^. The major advantage of this algorithm is that only predictor variables which significantly influence the response variable are included in the model. To further interpret the results from the SMLRM, we generated several continuous wavelet transformations (CWTs) from the hourly-measured CO_2_ flux time series and the residuals of the stepwise regression models. Finally, we calculated the Spearman correlation coefficients (ρ) between residual CO_2_ time series and fluid reinjection rates. In order to compare the two different time series, we reduced the hourly-measured CO_2_ fluxes to daily averages. This was done in two steps. We first used a 24-h Gaussian filter providing zero phase shift by running in both the forward and reverse directions, smoothing the hourly measured CO_2_ fluxes. As a second step, we used a shape-preserving piecewise cubic interpolation to identify adequate numbers of sampling points from the hourly-measured CO_2_ data and down sample the CO_2_ fluxes to the time series of the lower-resolution daily injection data (Fig. 2). Additionally, a linear regression analysis was performed to model the relationship between CO_2_ flux residuals and reinjection rates and calculate of a 95% confidence interval.

## Results and discussion

**Figure 3:**
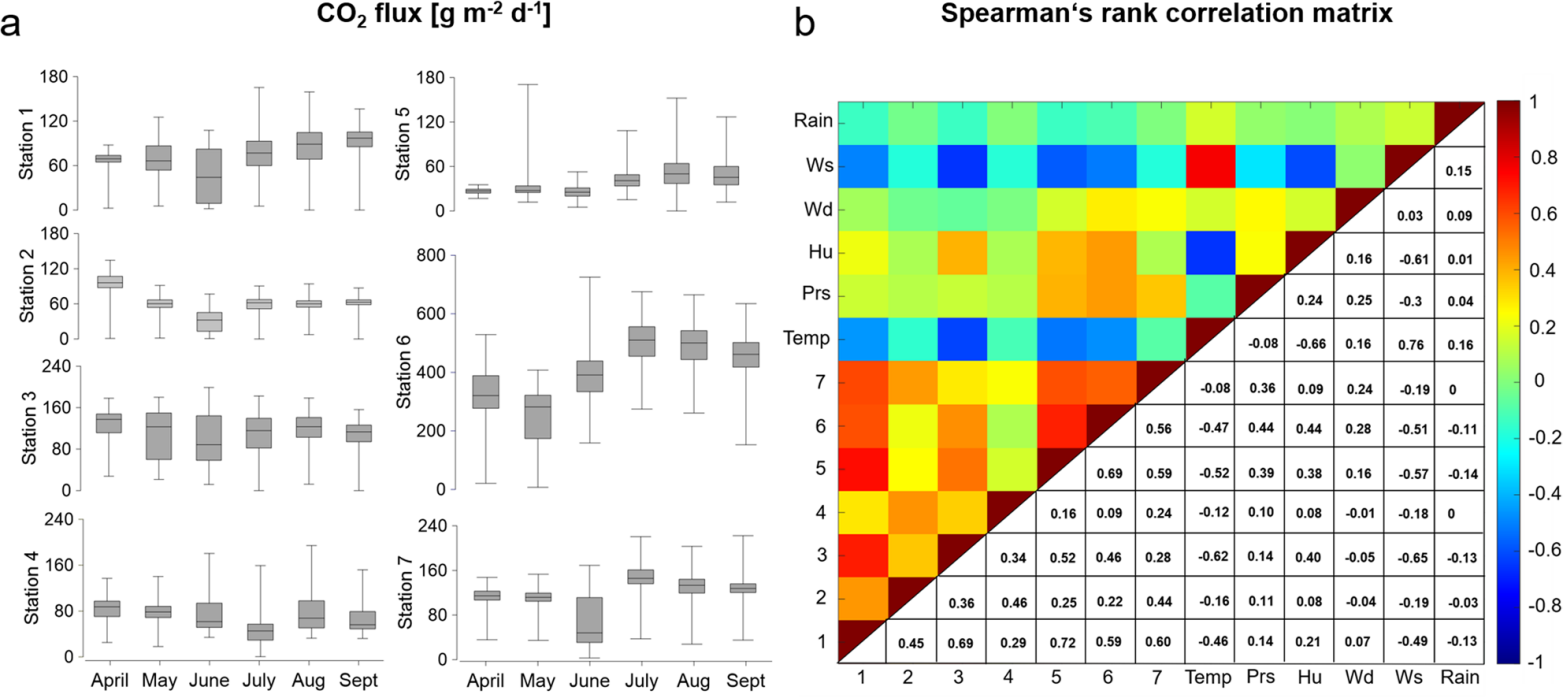
(**a**) Boxplots showing the variability of CO_2_ flux values during the monitoring period. (**b**) Spearman correlation coefficients showing the relationship between stations and atmospheric parameters. The y-axis labels are defined as follows: *Temp* air temperature, *Prs* barometric pressure, *Hu* air humidity, *Wd* wind direction, *Ws* wind speed, Rain.

Each CO_2_ emissions time series is characterized by strong variability in daily mean flux rates and a decrease at all stations from April to June, followed by a moderate increase from July to September (Fig. 3a, Fig. S2 and Table S2). Despite the close proximity of the seven monitoring stations, the different time series do not always indicate coherent behaviour. Given that background/biogenic CO_2_ fluxes at Los Humeros usually do not exceed 20 g m^−2^ d^−1^, the mean CO_2_ flux value of each station suggests input from hydrothermal degassing, as also supported by carbon isotopic samples taken at two sites within the monitoring area (δ^13^${\text{C}}_{{{\text{CO}}_{2} }}$ = − 3.3 and − 3.1‰)^44^. In fact, hydrothermal degassing at rates similar to biogenic fluxes can result from low permeability of soil/rocks or low-pressure gradients^31^. At Station 6, CO_2_ flux values were observed to be twice as high compared to values at the other monitoring stations. Along with a ground temperature of 97.2 °C, this is indicative of advective fluid transport. However, low degassing rates, as observed at station 2, 4 and 5, provide evidence for mixed diffusive-advective gas transport. Diurnal variations between 130–475 g m^−2^ d^−1^ (Station 6) demonstrate the dynamic behaviour of fluid migration within this highly-permeable fault zone. The strong variations of CO_2_ flux in such a constrained area are affected by (i) different transport mechanisms of fluid flow (advective/diffusive), (ii) variable intensities of hydrothermal alteration, (iii) subsurface heterogeneities, (iv) fault zone architecture/migration pathways, and eventually, (v) atmospheric parameters. Stations 1, 5, 6 and 7 demonstrate similar behavior to each other and to meteorological parameters (Fig. 3b).

### Atmospheric effects on CO_2_ flux

The Spearman´s rank correlation revealed that the strongest negative correlations occurred between CO_2_ flux and wind speed as well as air temperature. Both of these atmospheric parameters are strongly positively correlated with each other. Atmospheric pressure, on the other hand, shows only a weak positive correlation with CO_2_ flux at stations 5, 6, and 7, while at the other stations no correlation is detected.

Wind speed demonstrates the greatest influence on CO_2_ flux for the majority of stations (Table S4). The moderate to strong inverse correlation between wind speed and CO_2_ flux at stations 1, 3, 5, and 6, shown in blue (Fig. 3b), suggests that either high wind speeds inhibit the migration of CO_2_ from the soil or that CO_2_ is diluted with ambient air, that penetrates the shallow and partially porous subsurface favoured by topography. In fact, the west-facing topographic scarp of the NNE-SSW-striking Los Humeros fault is exposed to the main wind directions measured during the monitoring period (Fig. S4). A possible link between surface topography and CO_2_ flux has already been discussed in a study performed at Mammoth Mountain in California^45,46^. Considering that barometric pressure was relatively stable during the monitoring period (Fig. S3 and Table S3), we suspect that the positive correlation with CO_2_ flux (stations 5, 6 and 7) is either a spurious correlation or a superposition by stronger atmospheric parameters, such as wind speed, that masks the barometric pumping effect^47^.

Although rainfall and CO_2_ flux do not have significant correlation coefficients (Fig. 3b), we observe an effect of heavier rain periods on CO_2_ emissions (Fig. S2). We assume that with increased precipitation, the upper, altered soil layer becomes saturated with water, forming a gas seal that prevents CO_2_ degassing. To protect the equipment from condensation, no measurements were taken when air humidity exceeded 90%, resulting in a few data gaps during the end of June and in the first half of August. An overview of the statistical distribution of atmospheric parameters is provided in the supplementary material (Table S3).

Application of the SMLRM revealed that between 7 to 39% of CO_2_ flux variations can be explained by atmospheric parameters, with less than 10% of the variations explained by atmospheric parameters at stations 2 and 4. In comparison to the other stations, station 4 shows more spike-like variations and no cyclic behavior (Fig. S2, S5). A detailed summary of the results at each station can be found in Table S4 in the supplementary material.

**Figure 4:**
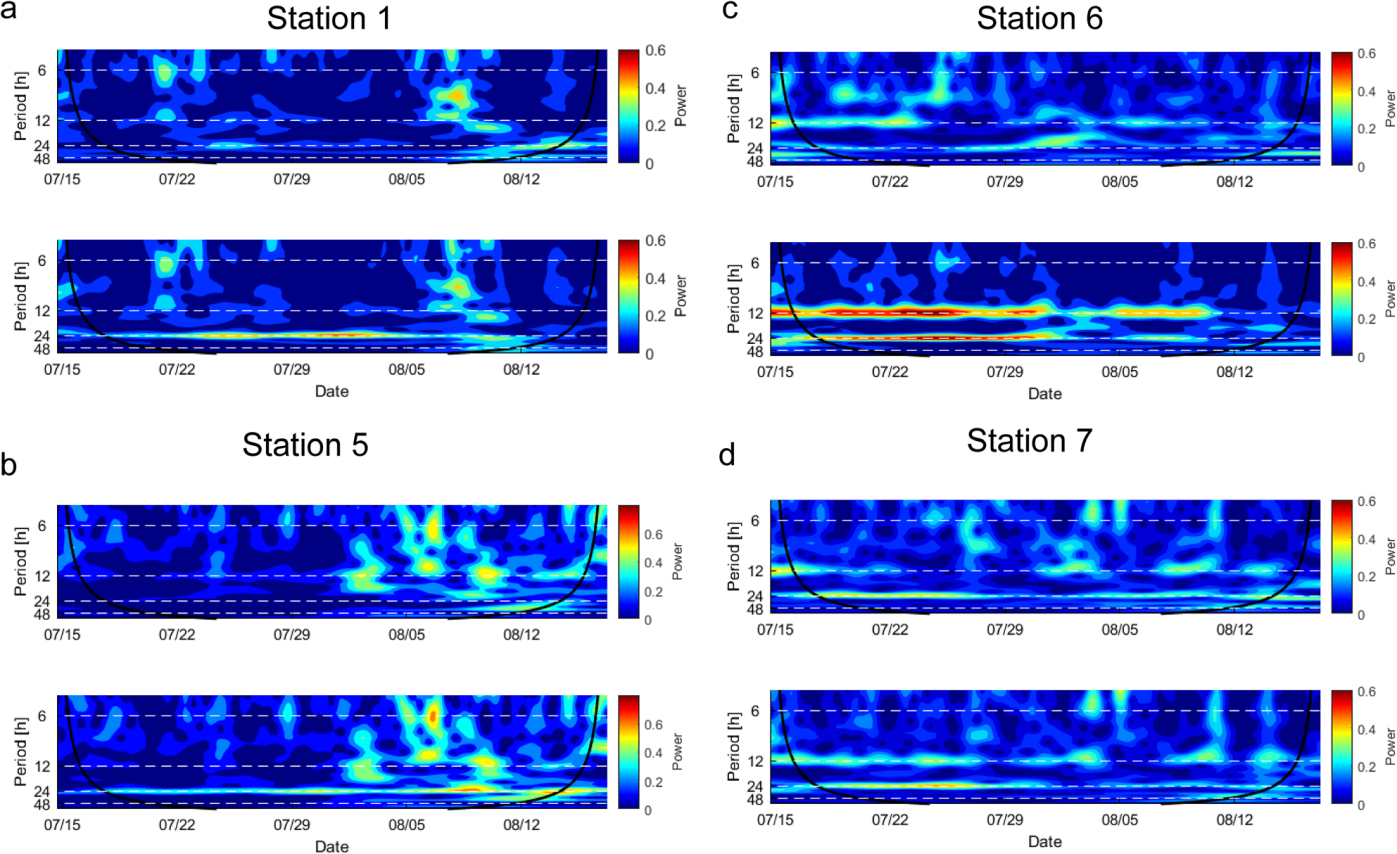
Wavelet power spectrum for the period from mid-July to mid-August at: (**a**) station 1, (**b**) station 5, (**c**) station 6, and (**d**) station 7. The lower spectrogram at each station shows the initial CO_2_ flux, while the upper spectrogram shows the residual CO_2_ flux. The time period shown here was chosen because of its continuous data coverage. Black solid lines represent the cone of influence, with areas outside the black line potentially affected by edge-effect artefacts. The wavelets were created with the MATLAB software, version R2019b.

The results of the CWT on CO_2_ flux and model residuals show that a 24-h cycle is evident at nearly all stations, while semi-diurnal cycles are less pronounced (Fig. 4, Fig. S5). It is not surprising that stations 2 and 4 do not show any cyclic behavior, as the results of the SMLRM show the least influence from atmospheric parameters, thus emphasizing the impact of unmonitored variables on their variations. The residual CO_2_ flux rates at stations 1 and 5 show no cyclic behavior, while for stations 3, 6 and 7, the strengths of 12-h and 24-h cycles become weaker but are still visible. Consequently, the SMLRM and CWT prove that further unmonitored variables affect CO_2_ flux variations, which are discussed in the following paragraph.

### Effects of the shallow subsurface on CO_2_ flux

Soil porosity and intrinsic permeability play major roles in the vadose zone, since they determine fluid flow mechanisms (advection/ diffusion) and flow directions^39,47,48^. On average, the uppermost layer in the geological succession of the Los Humeros geothermal reservoir consists of 100 m-thick, unconsolidated pumice and scoria fall deposits with porosities of up to 50%^29^. Hydrothermal alteration of varying intensity, as seen throughout the study area, is induced by fluid-rock interactions and affects petrophysical rock properties^26^.

Some studies have shown that soil temperature and soil water content contribute significantly to variations in CO_2_ flux due to increased biological oxidation, or near-surface steam condensation^47,49,50^, while others did not identify any significant relationship^45^. These parameters have not been measured continuously at our study site due to technical difficulties with the sensors. However, a strong correlation between ground temperatures and mean CO_2_ flux is supported by data from the initial site selection survey (e.g., station 1: 73 g m^−2^ d^−1^, 60.5 °C; station 3: 109 g m^−2^ d^−1^, 73.8 °C; station 6: 418 g m^−2^ d^−1^, 97.2 °C; and station 7: 120 g m^−2^ d^−1^, 57.3 °C; Fig. 1c) and reinforces the assumption that ground temperatures may explain some of the CO_2_ flux variation. Ultimately, the damage zone of the Los Humeros fault substantially influences fluid migration from the hydrothermal reservoir to the surface, as indicated by the strong variability of increased CO_2_ fluxes and hot ground temperatures. The increase in permeability of fault damage zones as a result of extensive fracture networks has previously been noted^49–54^. We relate heterogeneities and anisotropies in the shallow subsurface to a complex fracture network, acting as a fluid conduit-barrier system, with the geometry and distribution of fractures related to normal dip-slip kinematics and recent uplift of small magma bodies^55^. Mineral precipitation of quartz and calcite in fractures and faults is the result of silica-rich geothermal waters and loss of CO_2_ at the boiling point^33,56^. Together with hydrothermal alteration at the surface, these processes may impede lateral and vertical fluid migration in certain areas, while directing fluid flow to areas of higher permeability as previously observed in other geological systems^57^.

### CO_2_ flux vs. fluid reinjection—implication for geothermal reservoir management

**Figure 5:**
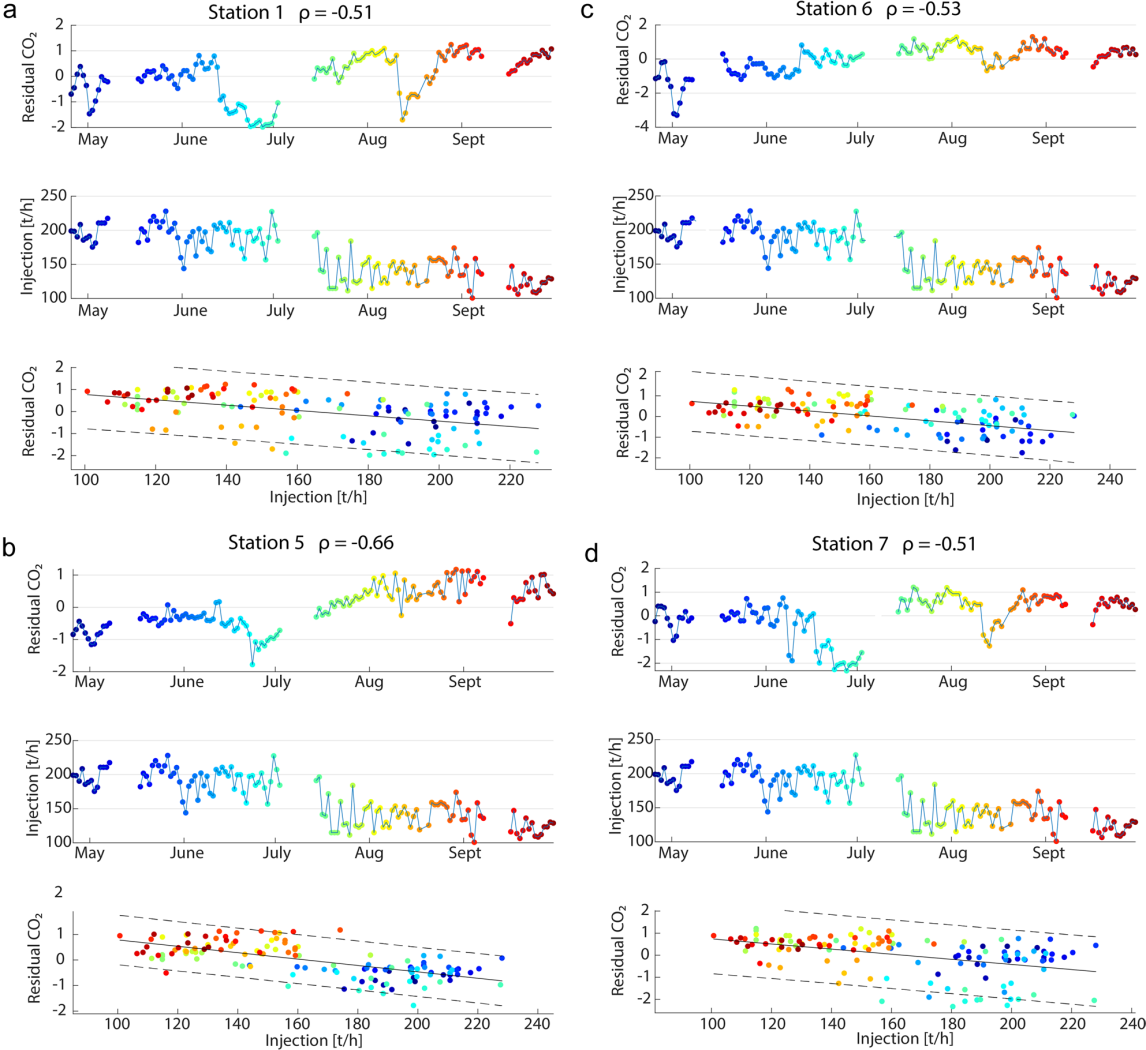
Temporal relationship between daily residual CO_2_ flux (upper plot) and total reinjected fluids (middle plot) at: (**a**) station 1, (**b**) station 5, (**c**) station 6, and (**d**) station 7 and corresponding spearman correlation coefficients (ρ). Linear regression analysis (bottom plots) illustrates the relationship between the CO_2_ flux residuals and reinjection rates within the 95% confidence interval. Colours are used to visualize the data of the respective time periods**.** The interrupted lines in the CO_2_ flux and reinjection curves represent data gaps that are not considered in the determination of correlation coefficients.

The key finding of this study is the inverse correlation between the rate of low-temperature (approx. 90 °C) reinjected fluids and residual CO_2_ flux (Fig. 5, Fig. S7). We identified this inverse correlation by removing the effects of measured atmospheric parameters and calculating Spearman’s rank correlation coefficients between both time series. For this purpose, we used the summarized flow rate of all three reinjection wells, referred to as total reinjected fluids (Fig. S6), and obtained moderate to strong negative correlation coefficients (ρ = − 0.51 to − 0.66) at stations 1, 5, 6, and 7 (Fig. 5). These stations are also intercorrelated and show intermediate to strong correlations with atmospheric parameters (Fig. 3b).

The spectral signatures of the residual time series from stations 1 and 5, visualized in their respective wavelet power spectra, no longer show diurnal variations (Fig. 4). These results demonstrate that the SMLRM successfully modelled atmospheric influences and that the residual time series are free of variations caused by atmospheric changes. Therefore, they may represent variability related to changes in the geothermal reservoir, which could explain the stronger correlation to reinjection of stations 1 and 5 compared to other stations. For example, the lowest mean CO_2_ flux (40 g m^−2^ d^−1^) of all stations was measured at station 5. However, a temporal relationship between low degassing rates, similar to those at station 5, and deep seismic activity has been monitored at Piton de la Fournaise^58^ and supports our finding that lower CO_2_ emissions can provide information about dynamic changes at depth. At stations 2, 3, and 4, no significant correlations to reinjection rates were calculated (Fig. S7). As mentioned earlier, stations 2 and 4 differed in their response to daily atmospheric variations. They showed strongly reduced power within the 24- and 12-h bands, indicating that those fluxes may not correspond as strongly to atmospheric changes as other stations (Fig. S6).

Station 3 shows strong 24- and 12-h cycles and a low correlation to reinjection rates of the respective residual CO_2_ flux time series. Comparing the wavelet power spectrum of station 3 with the wavelet power spectrum of its residuals, strong 24- and 12-h cycles remain, indicating that an atmospheric influence is still present (Fig. S6).

We assume that stations not correlated with reinjection rates are more strongly influenced by unmonitored variables, e.g., soil temperature or soil humidity, as well as nonlinear processes such as: (i) fluid-rock interactions (dissolution, mineralization) leading to changes in fracture permeability and soil/rock properties^33,59^, (ii) changes in effective stresses by pore pressure perturbations from ascending fluids^60^, and (iii) local and regional stress field changes^28,61^ due to volcanic-tectonic forces influencing fracture distribution and geometry.

It is therefore unreliable to consider only one parameter when trying to understand which processes are affecting CO_2_ in the subsurface, as complex physical, thermal, chemical and mechanical (THCM) processes occur during the reinjection of cold geothermal brine into geothermal systems^60–65^.

To understand the inverse correlation of surface CO_2_ emissions and reinjection of cold water, we will discuss some hypotheses below. However, we want to point out that none of these hypotheses is true on its own, but rather they become valid when combined.

1st hypothesis: The natural upflow of andesitic and fossil fluids from the deep volcanic system can be suppressed by high reinjection rates, reducing the ascendance of CO_2_ into the geothermal reservoir. This process has already been described by a numerical model reported in Ref.^63^.

2nd hypothesis: Ref.^63^ also determined that a large amount of non-condensable gases such as CO_2_ can be stored in reservoir rocks through mineral dissolution and precipitation.

Dissolved CO_2_ reacts with divalent cations such as Ca^2+^ and precipitates calcite, which is in agreement with hydrothermal zones composed of calcite and other hydrothermal minerals found in well cuttings at Los Humeros^56^. This trapping mechanism can be numerically modelled by either chemical or physical adsorption of gases on the rock matrix^63^.

3rd hypothesis: The deep reinjection of fluids into the low permeable rock matrix at 2000 m depth results on the one hand in a pressure buildup, causing CO_2_ to remain in the dissolved phase and on the other hand a reduction in boiling, which also has a positive effect on CO_2_ solubility^66^.

Future studies should focus on numerical models of coupled THCM processes, in order to evaluate the proposed hypotheses and the role of discrete fracture networks and multi-phase fluid flow.

However, the response of CO_2_ emissions to a decrease or increase in reinjection rates within 24 h indicates that the Los Humeros fault is a highly permeable structure, connecting the geothermal reservoir and Earth’s surface. To exclude a potential time delay between the response of CO_2_ emissions and fluid reinjection, we also calculated correlation coefficients when testing variable time lags, and did not observe an increase in correlation. Consequently, we can define the response time of gas emissions to changes in reinjection rates as ≤ 24 h. A global review paper on tracer tests summarizes that tracer velocities in the order of one to several tens of meters per hour are not exceptional^64^. Increased fluid migration velocities are also indicated by tracer studies performed in wells at Los Humeros^67^, thus supporting our results.

### Natural gas emissions vs. seismic activity

Induced seismicity triggered by geothermal exploitation causes changes in the thermal and poroelastic stresses of a reservoir^68^. During our monitoring period, recorded seismicity did not exceed a local magnitude of *M*_LV_ 2.1 (unpublished data) with the majority of hypocentres located at > 2 km depth, corresponding to the depth of the exploited geothermal reservoir^23^. In this study, we found no clear relationship between residual CO_2_ flux and seismicity rate or associated magnitudes. However, we suggest further study involving longer observation periods and seismic tremor analyses to validate this relationship. In addition, CO_2_ flux could be compared with more sensitive data such as structural changes obtained with coda wave interferometry^69^. For this purpose, we would place the gas monitoring system along the fault trace of seismically active faults with geothermal surface activity.

## Conclusion

**Figure 6:**
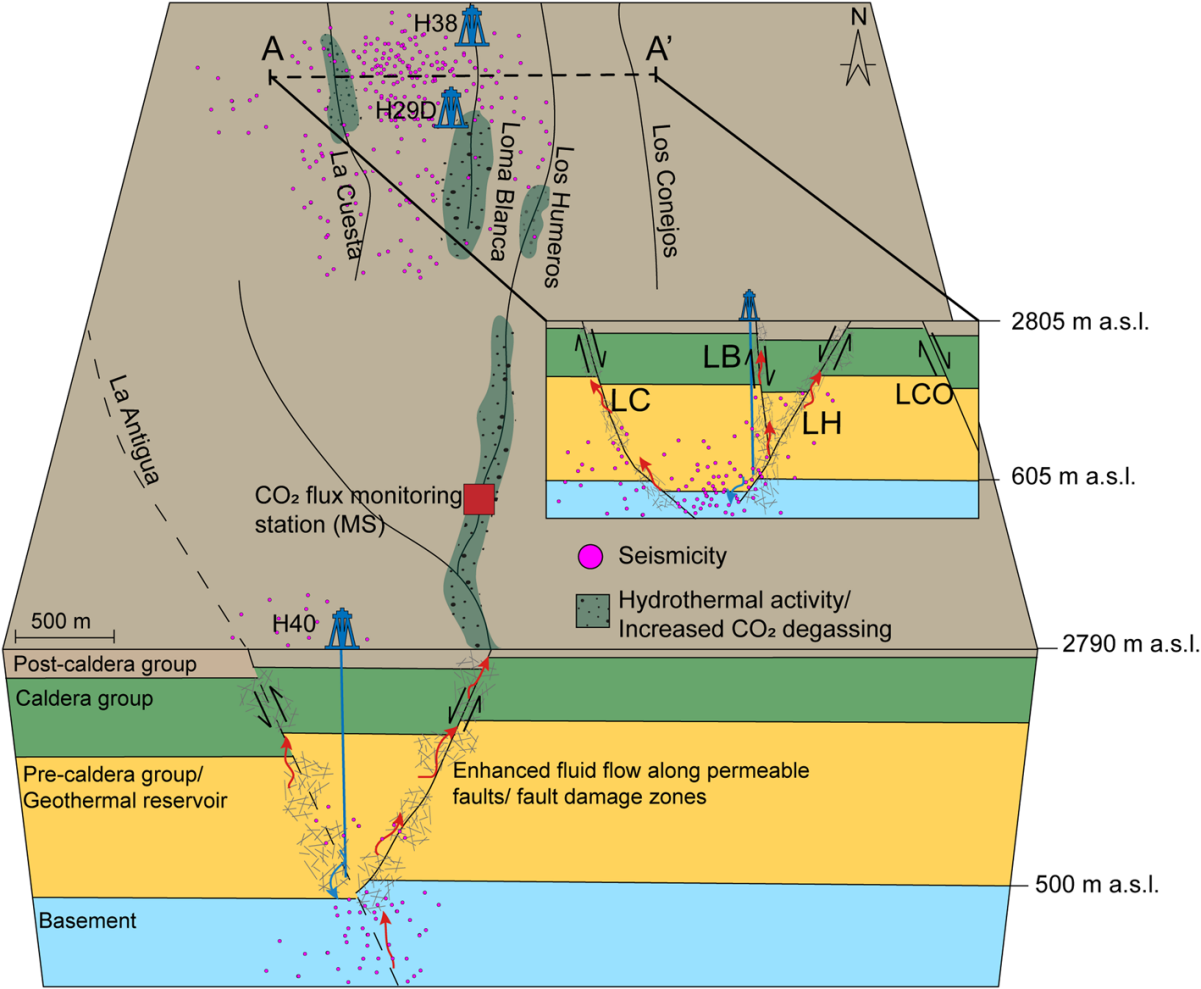
A simplified conceptual model showing enhanced fluid migration along steeply-dipping faults/fault damage zones cutting through the Los Humeros geothermal system. Cross sections show the orientation and connectivity of injection wells to faults. Red arrows illustrate the migration of hot reservoir fluids, while blue arrows show the migration of colder reinjected fluids. Note that cross section A–A′ is located between injection well H29D (deviated to the NW) and H38 and is intended to represent a buffer zone for this area. For the exact location of seismicity, the reader is referred to Fig. 1b. The abbreviations are defined as follows: *LH* Los Humeros, *LB* Loma Blanca, *LC* La Cuesta, *LCO* Los Conejos, *a.s.l*. above sea-level. Detailed descriptions of lithologies referring to the different caldera groups can be found in Ref.^70^.

The characterization of fluid migration in geothermal fields plays an important role for the safe and sustainable management of a reservoir. In this study, we have discussed various factors influencing the variation on CO_2_ emissions and demonstrated the effect of fluid reinjection on surface gas emissions. Our results indicate an active hydraulic communication between the target zones of reinjection wells and hydrothermal surface manifestations along the Los Humeros fault, as illustrated by a simplified conceptual model (Fig. 6). This finding has implications for novel reservoir monitoring concepts, including automated gas analytics for real-time analyses of reservoir responses to geothermal reservoir operations (including stimulations). Multi-chamber systems provide a fundamental tool for studying the high spatial and temporal variability of surface CO_2_ flux due to external factors, particularly within active structural settings where fluid flow is controlled by extensive fracture networks.

Several open questions remain; therefore future studies should investigate long-term monitoring of gas emissions (≥ 12 months, ideally 24 months) and multiple gas monitoring systems should be installed across geothermal fields. In addition, coupled THCM models considering discrete fracture networks, integrated multi-phase flow and reactive transport simulations could model the complexities discussed here, which is beyond the scope of this publication.

Nevertheless, we have provided important evidence that surface CO_2_ flux responds to reservoir-induced changes caused by reinjection of cold geothermal brine.

Regular sampling of carbon and noble gas isotopes using automated sampling techniques could complement the analysis of changing reservoir conditions driven by geothermal exploitation.

Geochemical and geophysical methods should be more commonly combined in integrated monitoring systems for optimized reservoir management. This also applies to other utilization concepts of the geological underground, such as carbon capture storage systems.

## Supplementary Information

Supplementary Information.

## Data Availability

A data publication is available for this paper at 10.5880/GFZ.4.8.2021.003.

## References

[1] Stelling P (2016). Geothermal systems in volcanic arcs: Volcanic characteristics and surface manifestations as indicators of geothermal potential and favorability worldwide. J. Volcanol. Geotherm. Res..

[2] Carrasco-Núñez G (2018). Reappraisal of Los Humeros volcanic complex by new U/Th Zircon and ^40^Ar/^39^Ar dating: Implications for greater geothermal potential. Geochem. Geophys. Geosyst..

[3] Suñe-Puchol I (2019). The Ilopango caldera complex, El Salvador: Origin and early ignimbrite-forming eruptions of a graben/pull-apart caldera structure. J. Volcanol. Geotherm. Res..

[4] Sorey ML (1985). Evolution and present state of the hydrothermal system in Long Valley Caldera. J. Geophys. Res..

[5] Amanda FF, Yamada R, Uno M, Okumura S (2019). Evaluation of caldera hosted geothermal potential during volcanism and magmatism in subduction system, NE Japan. Geofluids.

[6] Wohletz K, Grant H (1992). Volcanology and Geothermal Energy.

[7] Banerjee A (2011). Deep permeable fault-controlled helium transport and limited mantle flux in two extensional geothermal systems in the Great Basin, United States. Geology.

[8] Jolie E, Klinkmueller M, Moeck I, Bruhn D (2016). Linking gas fluxes at Earth’s surface with fracture zones in an active geothermal field. Geology.

[9] Werner C, Cardellini C (2006). Comparison of carbon dioxide emissions with fluid upflow, chemistry, and geologic structures at the Rotorua geothermal system, New Zealand. Geothermics.

[10] Taussi M (2021). Soil CO_2_ flux and temperature from a new geothermal area in the Cordón De Inacaliri volcanic complex (northern Chile). Geothermics.

[11] Chiodini G, Cioni R, Guidi M, Raco B, Marini L (1998). Soil CO_2_ flux measurements in volcanic and geothermal areas. Appl. Geochem..

[12] BatistaCruz RY (2019). Mantle degassing through continental crust triggered by active faults: The case of the Baja California Peninsula, Mexico. Geochem. Geophys. Geosyst..

[13] Kristmannsdóttir H, Ármannsson H (2003). Environmental aspects of geothermal energy utilization. Geothermics.

[14] Jolie E (2021). Geological controls on geothermal resources for power generation. Nat. Rev. Earth Environ..

[15] DiPippo R (2016). Geothermal Power Generation: Developments and Innovation.

[16] Stefansson V (1997). Geothermal reinjection experience. Geothermics.

[17] Kamila Z, Kaya E, Zarrouk SJ (2021). Reinjection in geothermal fields: An updated worldwide review 2020. Geothermics.

[18] Gaucher E (2015). Induced seismicity in geothermal reservoirs: A review of forecasting approaches. Renew. Sustain. Energy Rev..

[19] Horne RN (1985). Reservoir engineering aspects of reinjection. Geothermics.

[20] Cavazos-Álvarez JA, Carrasco-Núñez G (2020). Anatomy of the Xáltipan ignimbrite at Los Humeros Volcanic Complex; The largest eruption of the Trans-Mexican Volcanic Belt. J. Volcanol. Geotherm. Res..

[21] Negrín LCAG (2019). Current status of geothermal-electric production in Mexico. IOP Conf. Ser. Earth Environ. Sci..

[22] Carrasco-Núñez G (2017). Geologic map of Los Humeros volcanic complex and geothermal field, eastern Trans-Mexican Volcanic Belt. Terra Digit.

[23] Toledo T (2020). Local earthquake tomography at Los Humeros Geothermal Field (Mexico). J. Geophys. Res. Solid Earth.

[24] Elders WA, Izquierdo GM, Alfonso A, Tovar RA, Flores MA (2014). Significance of deep zones of intense bleaching and silicification in the Los Humeros high-temperature geothermal field, Mexico: Evidence of the effects of acid alteration. Trans. Geotherm. Resour. Counc..

[25] González-Partida E, Barragán RM, Nieva-G D (1993). Análisis geoquímico-isotópico de las especies carbónicas del fluido geotérmico de Los Humeros, Puebla, Mexico. Geofísica Int..

[26] Prol-Ledesma RM (1998). Pre- and post-exploitation variations in hydrothermal activity in Los Humeros geothermal field, Mexico. J. Volcanol. Geotherm. Res..

[27] Pinti DL (2017). Fluid circulation and reservoir conditions of the Los Humeros Geothermal Field (LHGF), Mexico, as revealed by a noble gas survey. J. Volcanol. Geotherm. Res..

[28] Norini G (2019). The structural architecture of the Los Humeros volcanic complex and geothermal field. J. Volcanol. Geotherm. Res..

[29] Weydt LM (2018). Outcrop analogue study to determine reservoir properties of the Los Humeros and Acoculco geothermal fields, Mexico. Adv. Geosci..

[30] Jentsch A (2020). Magmatic volatiles to assess permeable volcano-tectonic structures in the Los Humeros geothermal field, Mexico. J. Volcanol. Geotherm. Res..

[31] Peiffer L (2014). Fluid geochemistry and soil gas fluxes (CO_2_-CH_4_-H_2_S) at a promissory Hot Dry Rock Geothermal System: The Acoculco caldera, Mexico. J. Volcanol. Geotherm. Res..

[32] Raich J, Tufekcioglu A (2000). Vegetation and soil respiration: Correlations and controls. Biogeochemistry.

[33] Gutiérrez-Negrín, L. C. A. & Izquierdo-Montalvo, G. Review and update of the main features of the Los Humeros geothermal field, Mexico. in *World Geothermal Congress, Bali, Indonesia, 25–29 April 2010* (2010). 10.1016/j.bmc.2011.02.011.

[34] Dávila-Harris P, Carrasco-Núñez G (2014). An unusual syn-eruptive bimodal eruption: The Holocene Cuicuiltic Member at Los Humeros caldera, Mexico. J. Volcanol. Geotherm. Res..

[35] Cardellini C (2017). Monitoring diffuse volcanic degassing during volcanic unrests: The case of Campi Flegrei (Italy). Sci. Rep..

[36] Parkinson KJ (1981). An improved method for measuring soil respiration in the field. J. Appl. Ecol..

[37] Aragón-Aguilar A, Izquierdo-montalvo G, López-blanco S, Arellano-gómez V (2017). Analysis of heterogeneous characteristics in a geothermal area with low permeability and high temperature. Geosci. Front..

[38] Arellano VM (2015). The response to exploitation of the Los Humeros (México) Geothermal Reservoir. World Geotherm. Congr..

[39] Rinaldi AP, Vandemeulebrouck J, Todesco M, Viveiros F (2012). Effects of atmospheric conditions on surface diffuse degassing. J. Geophys. Res..

[40] Liuzzo M (2013). Ten years of soil CO_2_ continuous monitoring on Mt. Etna: Exploring the relationship between processes of soil degassing and volcanic activity. Geochem. Geophys. Geosyst..

[41] Cannata A (2010). Relationship between soil CO_2_ flux and volcanic tremor at Mt. Etna: Implications for magma dynamics. Environ. Earth Sci..

[42] Oliveira S, Viveiros F, Silva C, Pacheco JE (2018). Automatic filtering of soil CO_2_ flux data; different statistical approaches applied to long time series. Front. Earth Sci..

[43] Wilks DS (2006). Statistical Methods in the Atmospheric Sciences.

[44] Jentsch A (2020). Magmatic volatiles to assess permeable volcano-tectonic structures in the Los Humeros geothermal field, Mexico. J. Volcanol. Geotherm. Res..

[45] Lewicki JL, Hilley GE (2014). Multi-scale observations of the variability of magmatic CO_2_ emissions, Mammoth Mountain, CA, USA. J. Volcanol. Geotherm. Res..

[46] Lewicki JL (2007). Dynamic coupling of volcanic CO_2_ flow and wind at the Horseshoe Lake tree kill, Mammoth Mountain, California. Geophys. Res. Lett..

[47] Viveiros F, Gaspar JL, Guest JE, Duncan AM, Barriga FJAS, Chester DK (2015). Permanent monitoring of soil CO_2_ degassing at Furnas and Fogo volcanoes (São Miguel Island, Azores). Volcanic Geology of S. Miguel Island.

[48] Forde ON, Cahill AG, Beckie RD, Mayer KU (2020). Barometric-pumping controls fugitive gas emissions from a vadose zone natural gas release. Sci. Rep..

[49] Viveiros F (2020). Deep CO2 emitted at Furnas do Enxofre geothermal area (Terceira Island, Azores archipelago). An approach for determining CO_2_ sources and total emissions using carbon isotopic data. J. Volcanol. Geotherm. Res..

[50] Reth S, Reichstein M, Falge E (2005). The effect of soil water content, soil temperature, soil pH-value and the root mass on soil CO_2_ efflux—A modified model. Plant Soil.

[51] Bense VF, Gleeson T, Loveless SE, Bour O, Scibek J (2013). Fault zone hydrogeology. Earth-Sci. Rev..

[52] Caine JS, Evans JP, Forster CB (1996). Fault zone architecture and permeability structure. Geology.

[53] Rowland JV, Sibson RH (2004). Structural controls on hydrothermal flow in a segmented rift system, Taupo Volcanic Zone, New Zealand. Geofluids.

[54] Curewitz D, Karson JA (1997). Structural settings of hydrothermal outflow: Fracture permeability maintained by fault propagation and interaction. J. Volcanol. Geotherm. Res..

[55] Urbani S (2020). Estimating the depth and evolution of intrusions at resurgent calderas: Los Humeros (Mexico). Solid Earth.

[56] Martínez-Serrano RG (2002). Chemical variations in hydrothermal minerals of the Los Humeros geothermal system, Mexico. Geothermics.

[57] Yehya A, Rice JR (2020). Influence of fluid-assisted healing on fault permeability structure. J. Geophys. Res. Solid Earth.

[58] Boudoire G (2018). Small-scale spatial variability of soil CO_2_ flux: Implication for monitoring strategy. J. Volcanol. Geotherm. Res..

[59] Zhang Y (2008). Fault-related dilation, permeability enhancement, fluid flow and mineral precipitation patterns: Numerical models. Geol. Soc. Spec. Publ..

[60] Talwani P, Chen L, Gahalaut K (2007). Seismogenic permeability. J. Geophys. Res. Solid Earth.

[61] Norini G (2015). Structural analysis and thermal remote sensing of the Los Humeros Volcanic Complex: Implications for volcano structure and geothermal exploration. J. Volcanol. Geotherm. Res..

[62] Parisio F, Vilarrasa V, Wang W, Kolditz O, Nagel T (2019). The risks of long-term re-injection in supercritical geothermal systems. Nat. Commun..

[63] Kaya E, Zarrouk SJ (2017). Reinjection of greenhouse gases into geothermal reservoirs. Int. J. Greenh. Gas Control.

[64] Bödvarsson GS (1989). Some theoretical and field aspects of reinjection in geothermal reservoirs. Water Resour. Res..

[65] Bödvarsson GS, Tsang CF (1982). Injection and thermal breakthrough in fractures geothermal reservoirs. J. Geophys. Res..

[66] Pistone, S., Stacey, R. & Horne, R. The significance of CO_2_ Solubility in Geothermal Reservoirs. in *PROCEEDINGS, Thirty-Sixth Workshop on Geothermal Reservoir Engineering *(Stanford University, 2011).

[67] Iglesias, E. R. *et al.* Tracer testing at Los Humeros, Mexico, High-enthalpy geothermal field. in *Proceedings World Geothermal Congress 2015, Melbourne, Australia, 19–25 April 2015* (2015).

[68] Urban E, Lermo J (2017). Fracture and stress evaluation using well logs and microseismicity, in the exploitation of Los Humeros geothermal field, Mexico. Trans. Geotherm. Resour. Counc..

[69] Obermann A, Kraft T, Larose E, Wiemer S (2015). Potential of ambient seismic noise techniques tomonitor the St. Gallen geothermal site (Switzerland). J. Geophys. Res. Solid Earth.

[70] Carrasco-Núñez G, López-Martínez M, Hernández J, Vargas V (2017). Subsurface stratigraphy and its correlation with the surficial geology at Los Humeros geothermal field, eastern Trans-Mexican Volcanic Belt. Geothermics.

