# Supplementary material for: Monitoring the response of volcanic CO2 emissions to changes in the Los Humeros hydrothermal system
Source: Sci Rep. 2021 Sep 9;11:17972. doi: 10.1038/s41598-021-97023-x (PMC8429443; doi:10.1038/s41598-021-97023-x)
Supplement: Supplementary file 1 — Supplementary Information. [file 41598_2021_97023_MOESM1_ESM.docx]

**Supplementary Materials for: Monitoring the response of volcanic CO_2_ emissions to changes in the Los Humeros hydrothermal system**

Anna Jentsch, Walter Duesing, Egbert Jolie and Martin Zimmer

Correspondence to: ajentsch@gfz-potsdam.de

**This PDF file includes:**

Tables S1 - S4

Figures S1 – S7

TABLE S1. TECHNICAL DATA OF SENSORS FROM THE MWS 9-5 MICROPROCESSOR WEATHER STATION

| Variable | Sensor Type | Range | Accuracy |
| --- | --- | --- | --- |
| Temperature | PT-100 (Platinum resistance) | -40 to +50 °C | ± 0.3 °C |
| Barometric pressure | monolithic, laser trimmed absolute pressure sensor (thick film ceramic) | 600 to 1100 hPa | ± 0.8 hPa  (btw. -40 to 50°C) |
| Humidity | Monolithic, capacitive sensor | 10-100 % | ± 2 %  (btw. -40 to 50°C) |
| Windspeed | Three-shell anemometer with magnetic scanning measuring contactless using a Hall sensor (V1.2) or a Reed-contact. | 0 to 150 km/h | ± 2 km/h |
| Rain | Water is collected on an area of 200 cm^2^ and is drained through a funnel to the bucket. The bucket tilts whenever a defined quantity of water has been collected. The tilting creates pulses which are counted. | 0 to 5000 mm (0.1mm resolution) | ± 0.2 mm |
| Wind direction | A weather vane with an endless precision potentiometer of 10 kOhms and a rotation angle of 360 ° | 0 to 359,9 °N, (resolution 0.1°) | ± 5°  (at -10°C to 50°C), Hysteresis < 8° |
|  |  |  |  |

TABLE S2. DESCRIPTIVE STATISTICS OF CO_2_ FLUX FROM SEVEN MONITORING STATIONS

| Station | Minimum  (g m^-2^d^-1^) | Mean  (g m^-2^d^-1^) | Median  (g m^-2^d^-1^) | Maximum  (g m^-2^d^-1^) | SD | No. of data |
| --- | --- | --- | --- | --- | --- | --- |
| 1 | 1.9 | 73.7 | 79.7 | 165.4 | 30.2 | 2941 |
| 2 | 0.8 | 55.9 | 59.7 | 143.2 | 21.2 | 2167 |
| 3 | 11.6 | 109.5 | 117.1 | 198.8 | 39.1 | 2399 |
| 4 | 0.4 | 69.0 | 63.0 | 349.3 | 33.4 | 2602 |
| 5 | 5 | 40 | 36 | 170.6 | 17.5 | 2463 |
| 6 | 7.6 | 418.4 | 437.6 | 725.5 | 119.8 | 2640 |
| 7 | 3 | 120 | 125.3 | 222.3 | 36.4 | 2669 |
| Note: Soil CO_2_ flux was measured with a Licor-8100A automated monitoring system with 20 cm survey chambers. The analyzer control unit consists of an infrared gas analyzer and a connection panel to interface with monitoring chambers and the LI-8150 Multiplexer measuring the gas exchange. Measurement Range CO_2_: 0 ppm to 20,000 ppm; Accuracy: 1.5% of reading; SD - Standard Deviation. | | | | | | |

TABLE S3. DESCRIPTIVE STATISTICS OF ATMOSPHERIC PARAMETERS

| Variable | Minimum | Mean | Median | Maximum | SD | No. of data |
| --- | --- | --- | --- | --- | --- | --- |
| Atmospheric temperature  (°C) | 1.8 | 12.6 | 11.0 | 27.3 | 5.5 | 2974 |
| Barometric pressure  (hPa) | 728.1 | 731.9 | 731.8 | 735.7 | 1.4 | 2975 |
| Humidity  (%) | 10.7 | 74.6 | 82.0 | 98.1 | 20.5 | 2975 |
| Windspeed  (km/h) | 0 | 3.8 | 1.5 | 21.3 | 4.5 | 2975 |
| Rain  (mm) | 0 | 0.13 | 0 | 16.54 | 0.65 | 3048 |
| Wind direction  (°N) | 0 | - | - | 349.6 | - | 2428 |
| SD – Standard Deviation. | |  |  |  |  |  |

Table S4. RESULTS FROM THE STEPWISE MULTIPLE LINEAR REGRESSION ANALYSIS FOR EACH STATION

| Station | Estimated coefficients | Estimate | SE | tStat | pValue | Adjusted R² |
| --- | --- | --- | --- | --- | --- | --- |
| 1 | Intercept | -0.19 | 0.03 | -6.69 | 2.69e-11 |  |
|  | Air temperature | -0.16 | 0.04 | -3.63 | 2.83E-04 | 0.0165 |
|  | Barometric pressure | 0.09 | 0.02 | 4.9 | 1.04e-06 | 0.004 |
|  | Humidity | -0.2 | 0.04 | -5.4 | 7.21e-08 | 0.0311 |
|  | Wind speed | -0.30 | 0.03 | -9.24 | 4.48e-20 | 0.0544 |
|  | Rain | -0.08 | 0.02 | -4.32 | 1.61e-05 | 0.001 |
|  | Temp: Prs | 0.09 | 0.03 | 2.89 | 0.003 | 0.002 |
|  | Temp: Hu | -0.12 | 0.03 | -4.36 | 1.35e-05 | 0.012 |
|  | Temp: Ws | 0.30 | 0.05 | 5.97 | 2.66e-09 | 0.003 |
|  | Temp: Rain | 0.08 | 0.03 | 2.31 | 0.02 | 0.015 |
|  | Prs: Hu | 0.11 | 0.03 | 4.18 | 2.95e-05 | 0.002 |
|  | Prs: Ws | 0.08 | 0.03 | 2.99 | 0.002 | 0.006 |
|  | Ws: Rain | 0.06 | 0.03 | 2.22 | 0.03 | 0.007 |
| Linear regression model | y ~ 1 + Temp*Prs + Temp*Hmdt + Temp*Ws + Temp*Prcpt + Prs*Hmdt + Prs*Ws + Hmdt*Ws + Ws*Prcpt | | | | |  |
| Model summary statistics | Sum adjusted R² | 0.15 |  |  |  |  |
|  | Number of observations | 2971 |  |  |  |  |
|  | P-value | 2.93e-95 |  |  |  |  |
| Note: The gray marked row highlights the atmospheric variable that has the strongest influence on the CO2 flux. CO_2_ flux. SE – Standard Error; tStat - t-statistic for each coefficient to test the null hypothesis that the corresponding coefficient is zero against the alternative that it is different from zero, given the other predictors in the model; pValue - p-value for the t-statistic of the hypothesis test that the corresponding coefficient is equal to zero or not; Adjusted R² - The model explains the variability on the response variable (CO_2_ flux) caused by the predictor variables (atmospheric parameters). | | | | | | |

| Station | Estimated coefficients | Estimate | SE | tStat | pValue | Adjusted R² |
| --- | --- | --- | --- | --- | --- | --- |
| 2 | Intercept | -0.06 | 0.02 | -2.48 | 0.01 |  |
|  | Air temperature | -0.3 | 0.03 | -8.99 | 4.39E-19 | 0.0234 |
|  | Barometric pressure | 0.12 | 0.02 | 6.39 | 1.92e-10 | 0.0126 |
|  | Humidity | -0.36 | 0.03 | -11.34 | 3.24e-29 | 0.0226 |
|  | Rain | -0.05 | 0.02 | -2.3 | 0.02 | 0.0011 |
|  | Temp: Hu | -0.06 | 0.02 | -3 | 0.002 | 0.0033 |
|  | Temp: Rain | 0.13 | 0.03 | 4.23 | 2.4e-05 | 0.0038 |
|  | Prs: Rain | 0.05 | 0.02 | 2.26 | 0.02 | 0.0012 |
| Linear regression model | y ~ 1 + Temp*Hmdt + Temp*Prcpt + Prs*Prcpt | | | | |  |
| Model summary statistics | Adjusted R² | 0.07 |  |  |  |  |
|  | Number of observations | 2971 |  |  |  |  |
|  | P-value | 4.7e-45 |  |  |  |  |

| Station | Estimated coefficients | Estimate | SE | tStat | pValue | Adjusted R² |
| --- | --- | --- | --- | --- | --- | --- |
| 3 | Intercept | 0.06 | 0.02 | 3.09 | 2.01E-03 |  |
|  | Air temperature | -0.49 | 0.03 | -15.16 | 4.72E-50 | 0.009 |
|  | Barometric pressure | 0.09 | 0.02 | 5.51 | 3.85E-08 | 0.008 |
|  | Humidity | -0.30 | 0.03 | -11.69 | 6.98E-31 | 0.04 |
|  | Wind speed | -0.34 | 0.03 | -13.53 | 1.54E-40 | 0.304 |
|  | Rain | -0.07 | 0.02 | -4.64 | 3.65E-06 | 0.001 |
|  | Temp: Hu | -0.14 | 0.02 | -6.35 | 2.54E-10 | 0.005 |
|  | Prs: Hu | 0.03 | 0.02 | 1.79 | 7.33E-02 | 0.001 |
|  | Prs: Ws | 0.10 | 0.02 | 5.57 | 2.83E-08 | 0.003 |
|  | Hu: Ws | 0.16 | 0.02 | 7.82 | 7.48E-15 | 0.004 |
|  |  |  |  |  |  |  |
| Linear regression model | y ~ 1 + Temp*Hmdt + Prs*Hmdt + Prs*Ws + Hmdt*Ws + Ws*Prcpt | | | | |  |
| Model summary statistics | Adjusted R² | 0.39 |  |  |  |  |
|  | Number of observations | 2971 |  |  |  |  |
|  | P-value | 2.94e-309 |  |  |  |  |

| Station | Estimated coefficients | Estimate | SE | tStat | pValue | Adjusted R² |
| --- | --- | --- | --- | --- | --- | --- |
| 4 | Intercept | 0.00 | 0.02 | 0.15 | 8.80E-01 |  |
|  | Air temperature | -0.30 | 0.04 | -8.39 | 7.21E-17 | 0.0192 |
|  | Barometric pressure | 0.19 | 0.02 | 10.34 | 1.17E-24 | 0.0251 |
|  | Wind speed | -0.29 | 0.03 | -9.72 | 5.48E-22 | 0.0019 |
|  | Humidity | -0.10 | 0.03 | -3.79 | 1.51E-04 | 0.0304 |
|  | Prs: Hu | 0.05 | 0.02 | 2.85 | 4.45E-03 | 0.0016 |
| Linear regression model | y ~ 1 + Temp + Ws + Prs*Hmdt | | | | |  |
| Model summary statistics | Sum adjusted R² | 0.08 |  |  |  |  |
|  | Number of observations | 2971 |  |  |  |  |
|  | P-value | 2.95e-55 |  |  |  |  |

| Station | Estimated coefficients | Estimate | SE | tStat | pValue | Adjusted R² |
| --- | --- | --- | --- | --- | --- | --- |
| 5 | Intercept | -0.19 | 0.02 | -8.13 | 6.49E-16 |  |
|  | Air temperature | -0.10 | 0.03 | -4.05 | 5.16E-05 | 0.009 |
|  | Barometric pressure | 0.22 | 0.02 | 12.75 | 2.79E-36 | 0.031 |
|  | Wind speed | -0.33 | 0.03 | -11.67 | 8.68E-31 | 0.101 |
|  | Temp: Prs | 0.03 | 0.02 | 2.06 | 3.92E-02 | 0.01 |
|  | Temp: Ws | 0.22 | 0.02 | 9.73 | 4.96E-22 | 0.029 |
|  |  |  |  |  |  |  |
| Linear regression model | y ~ 1 + Temp*Prs + Prs*Hmdt + Prs*Ws + Hmdt*Ws | | | | |  |
| Model summary statistics | Adjusted R² | 0.18 |  |  |  |  |
|  | Number of observations | 2971 |  |  |  |  |
|  | P-value | 3.89e-120 |  |  |  |  |

| Station | Estimated coefficients | Estimate | SE | tStat | pValue | Adjusted R² |
| --- | --- | --- | --- | --- | --- | --- |
| 6 | Intercept | 0.00 | 0.02 | -0.10 | 9.21E-01 |  |
|  | Air temperature | 0.10 | 0.03 | 2.95 | 3.22E-03 | 0.003 |
|  | Barometric pressure | 0.18 | 0.02 | 10.28 | 2.24E-24 | 0.045 |
|  | Humidity | 0.18 | 0.03 | 5.71 | 1.25E-08 | 0.012 |
|  | Wind speed | -0.28 | 0.03 | -10.70 | 3.05E-26 | 0.138 |
|  | Temp: Prs | -0.08 | 0.03 | -2.71 | 6.85E-03 | 0.001 |
|  | Prs: Hu | -0.20 | 0.02 | -8.06 | 1.06E-15 | 0.018 |
|  | Prs: Ws | -0.08 | 0.02 | -3.48 | 5.05E-04 | 0.01 |
|  | Hu: Ws | 0.12 | 0.02 | 6.95 | 4.40E-12 | 0.012 |
| Linear regression model | y ~ 1 + Temp*Prs + Prs*Hmdt + Prs*Ws + Hmdt*Ws | | | | |  |
| Model summary statistics | Adjusted R² | 0.239 |  |  |  |  |
| Number of observations | 2971 |  |  |  |  |  |
|  | P-value | 2.29e-171 |  |  |  |  |

| Station | Estimated coefficients | Estimate | SE | tStat | pValue | Adjusted R² |
| --- | --- | --- | --- | --- | --- | --- |
| 7 | Intercept | -0.28 | 0.03 | -10.39 | 7.22E-25 |  |
|  | Air temperature | -0.08 | 0.04 | -2.01 | 4.46E-02 | 0.0113 |
|  | Barometric pressure | 0.25 | 0.02 | 13.00 | 1.33E-37 | 0.0416 |
|  | Humidity | -0.18 | 0.03 | -5.50 | 4.03E-08 | 0.0241 |
|  | Wind speed | -0.09 | 0.03 | -2.75 | 6.07E-03 | 0.024 |
|  | Temp: Ws | 0.40 | 0.05 | 7.81 | 7.63E-15 | 0.0215 |
|  | Prs: Ws | 0.06 | 0.02 | 3.48 | 5.13E-04 | 0.004 |
|  | Hu: Ws | 0.13 | 0.04 | 3.54 | 4.04E-04 | 0.003 |
| Linear regression model | y ~ 1 + Temp*Ws + Prs*Ws + Hmdt*Ws | | | | |  |
| Model summary statistics | Adjusted R² | 0.113 |  |  |  |  |
|  | Number of observations | 2971 |  |  |  |  |
|  | P-value | 3.72e-74 |  |  |  |  |

**
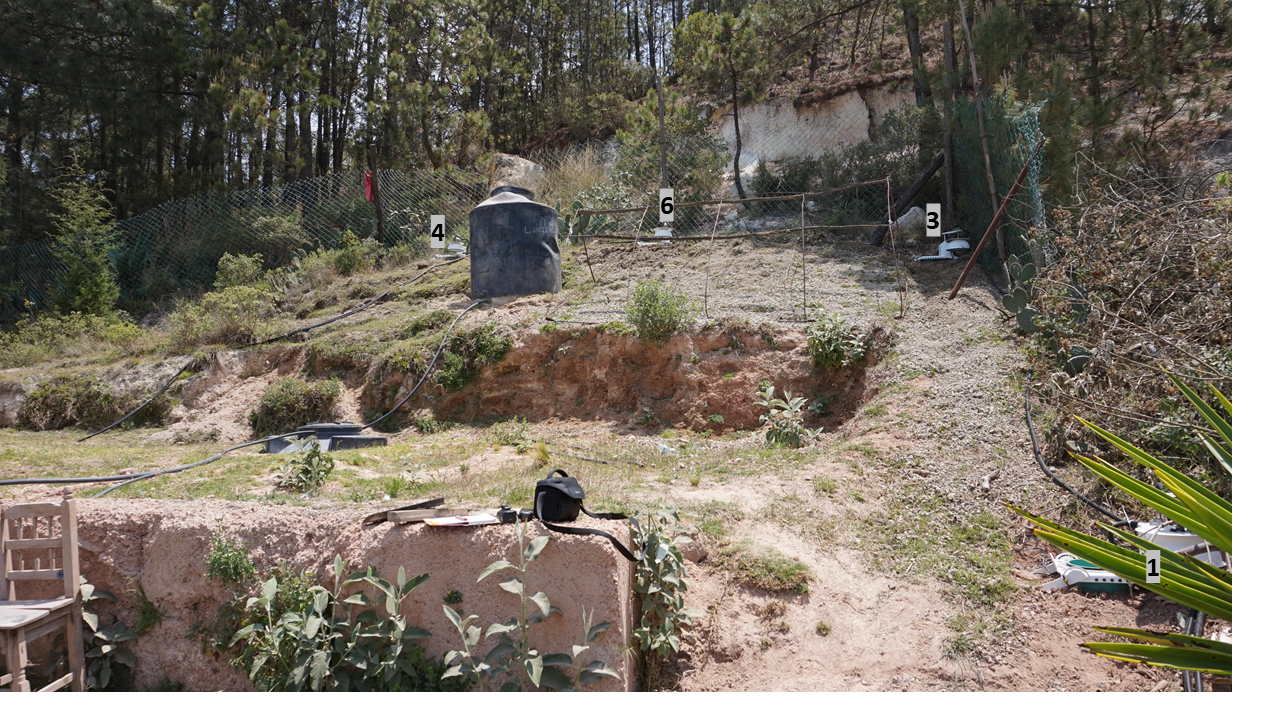
**

**Figure S1. P**hotograph of the study area showing four of the seven chambers. Numbers indicate the stations. Photograph is looking to the east towards the Los Humeros fault plane striking NNE-SSW.


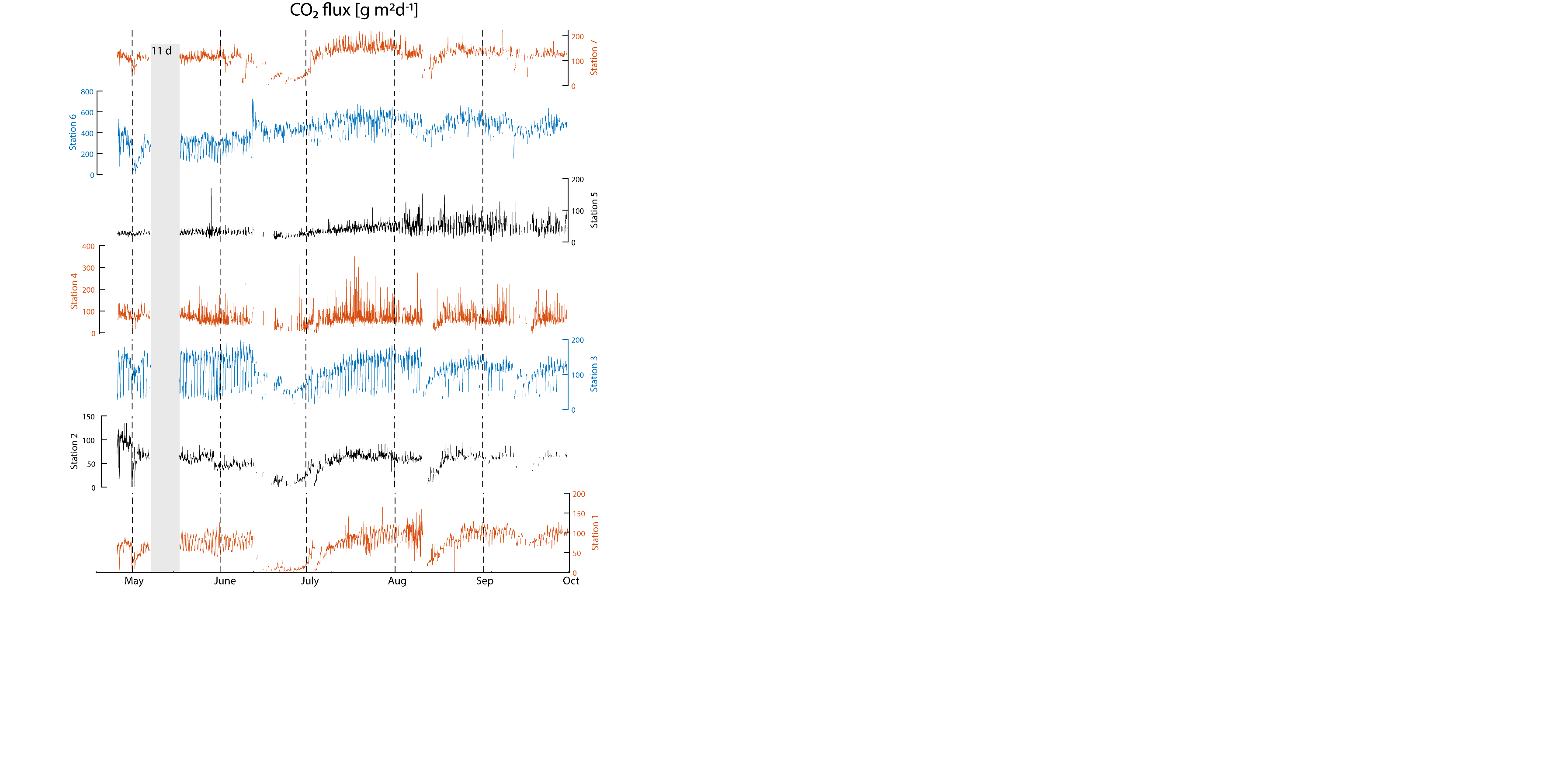


**Figure S2.** Temporal evolution of CO_2_ fluxes at all Stations from end of April to end of September 2018. Grey rectangle represents a data gap of 11 days. Black dashed vertical lines indicate the beginning of each month.


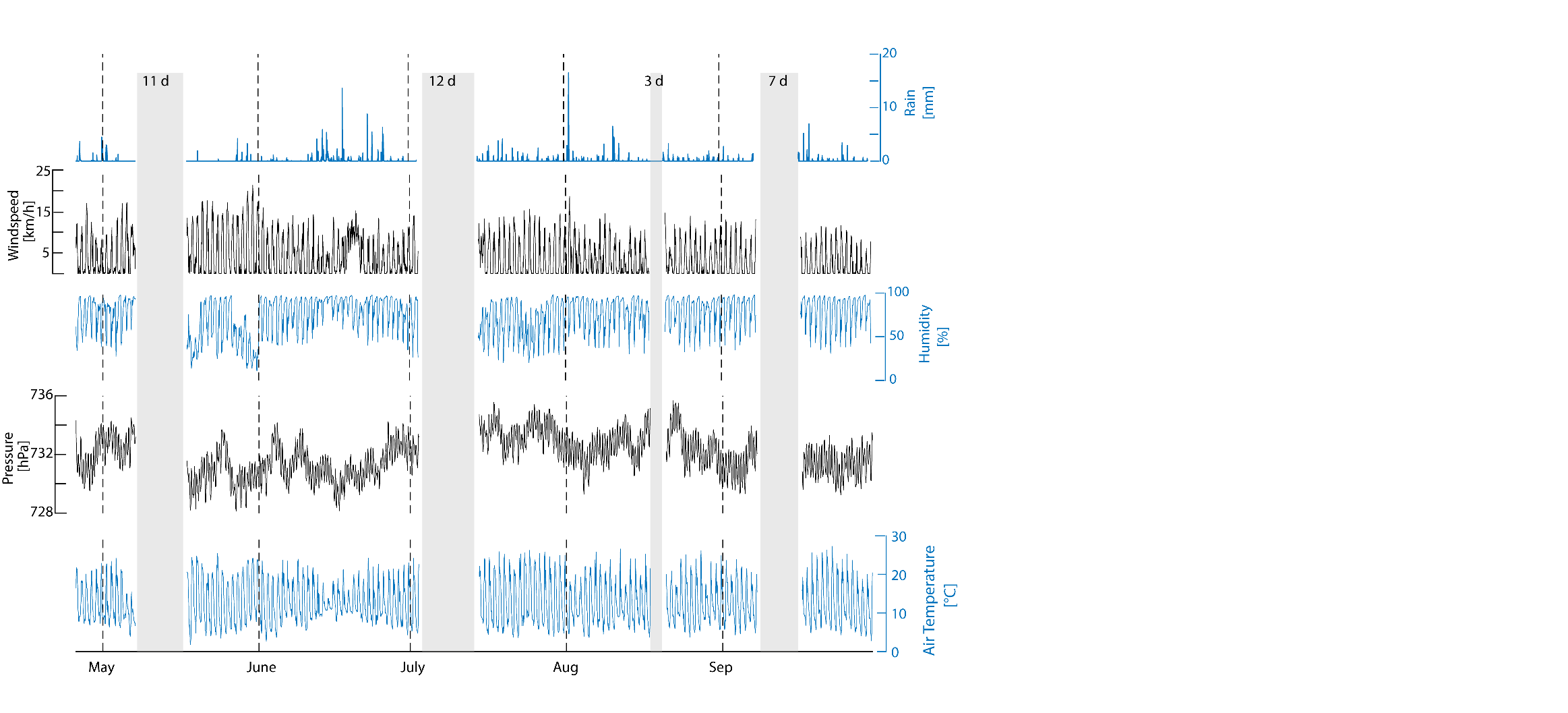


**Figure S3.** Temporal evolution of atmospheric parameters from end of April to end of September 2018. Grey rectangles represent the length of data gaps in days. Black dashed vertical lines indicate the beginning of each month.


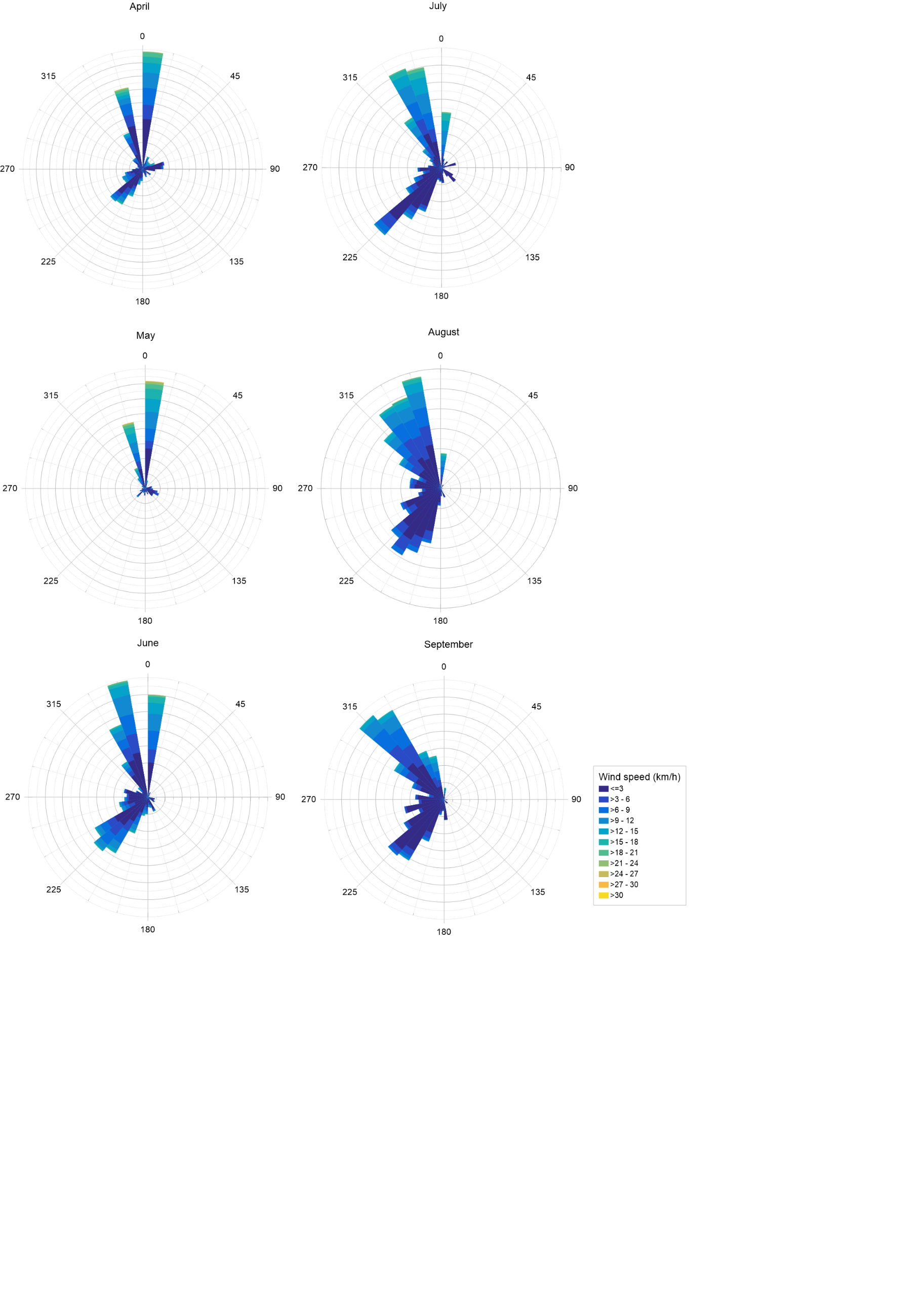


**Figure S4.** Windspeed and wind direction over the course of the monitoring period.


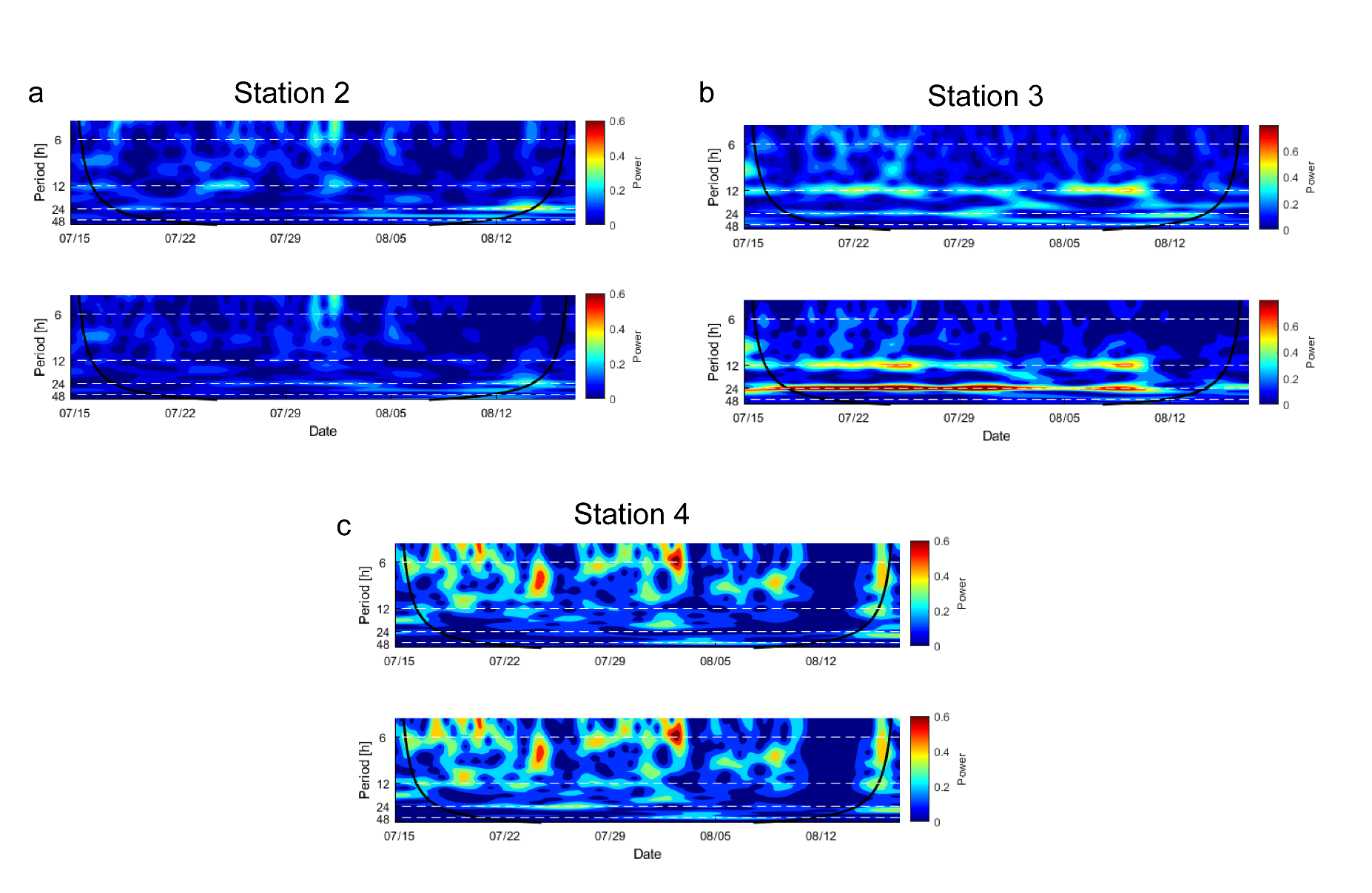


**Figure S5.** Wavelet power spectrum for the period from mid-July to mid-August at a) Station 2, b) Station 3, and c) Station 4. The lower spectrogram at each station shows the original CO_2_ flux while the upper wavelet power spectrum always shows residual CO_2_ flux. The time period shown here was chosen because of its continuous data coverage. Black solid lines represent the cone of influence with areas outside the black line potentially affected by edge effect artifacts.

**Figure S6.** Daily reinjection rates for each well and total rate of reinjected fluid (black). For the location of reinjection wells please see figure 1b


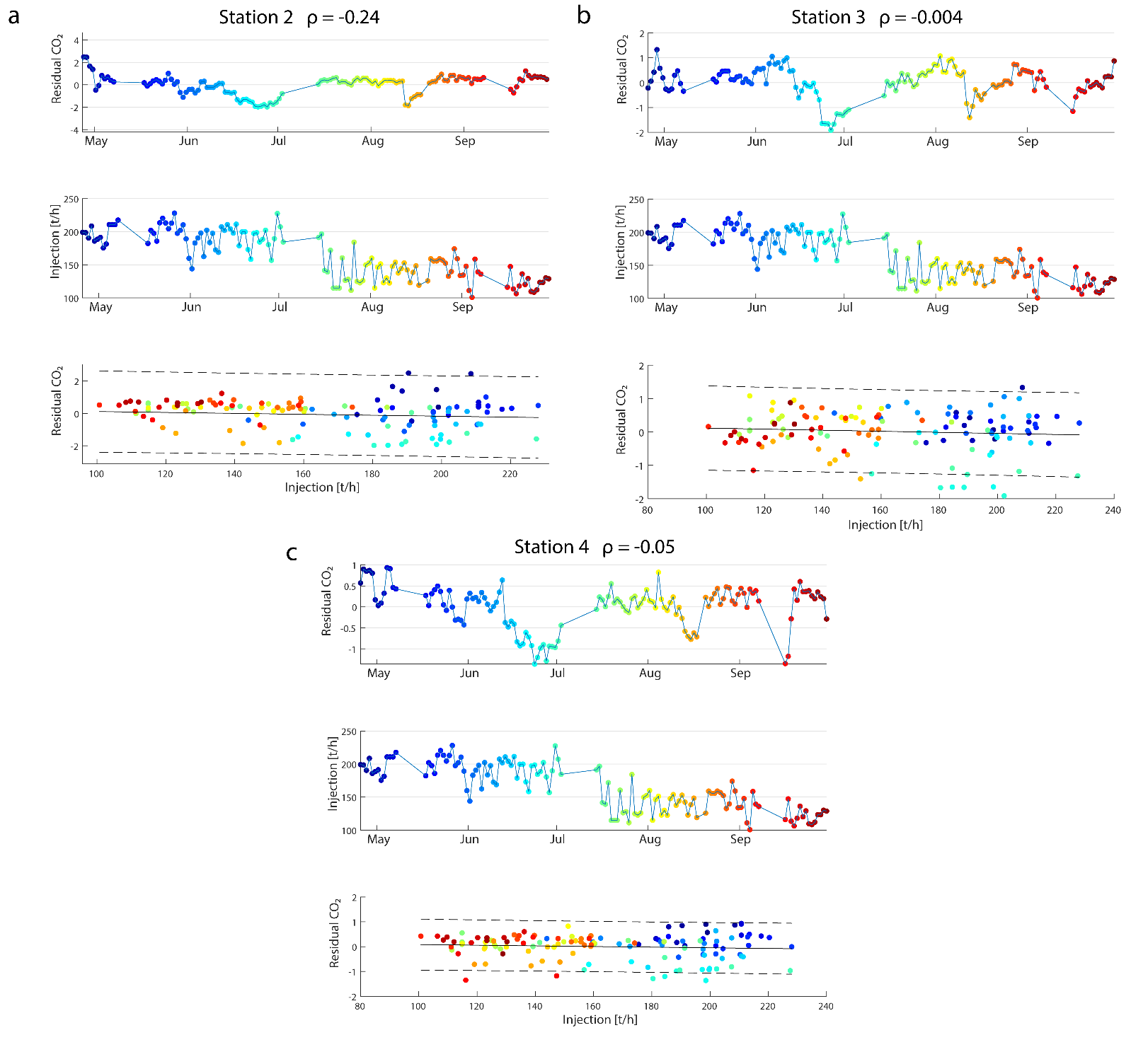


**Figure S7.** Temporal relationship between daily residual CO_2_ flux (upper plot) and total reinjected fluids (middle diagram) at: a) station 2, b) station 3, and c) station 4 and corresponding spearman correlation coefficients (ρ). Linear regression analysis (bottom plot) illustrates the relationship between the CO_2_ flux residuals and reinjection rates within the 95% confidence interval. Colours are used to visualize the data of the respective time period.
